# Cell cycle re‐entry primes neuronal senescence in brain aging and dementia

**DOI:** 10.1002/alz.095808

**Published:** 2025-01-09

**Authors:** Hei‐Man Chow

**Affiliations:** ^1^ The Chinese University of Hong Kong, Hong Kong Hong Kong

## Abstract

**Background:**

Emerging evidence strongly suggests that terminally differentiated neurons in the brain have the potential to undergo a cell cycle‐like process during neuronal aging and in the presence of certain diseases. However, due to their infrequent occurrence and unpredictable distribution within the brain, the molecular characteristics and specific variations associated with these cells in different diseases are still not well understood.

**Method:**

By taking advantage of the wealth of human brain single‐nucleus RNA sequencing (snRNA‐seq) datasets available in public repositories, we developed an analytical pipeline that facilitates the identification and characterization of cell cycle gene re‐expressing neurons to address these questions. The cell cycle gene expression status of each single nucleus was identified and subsequently tested for DNA duplication or deletion events might be due to aberrant cell cycle activity. Subsequently, the target cells of interest were further characterized via cell fate trajectory analysis to uncover their origins and evolutionary relationships. Lastly, to understand the potential involvement of these cells in disease development and heterogeneity, we quantitatively analyzed their relative numbers and performed differential gene expression analysis comparing nuclei from control and disease‐affected samples.

**Result:**

Our analysis showed that cell cycle‐related events primarily occur in excitatory neurons, with cellular senescence being their likely end fate. The number of neurons re‐engaging in the cell cycle and undergoing senescence decreased during normal brain aging, but in late‐onset Alzheimer’s disease (LOAD), these cells accumulated instead. Transcriptomic profiling of these cells revealed that disease‐specific differences were predominantly associated with early‐stage senescence, indicating increased proinflammatory, metabolic dysregulation, and pathology‐related signatures in diseased brains. Similar features were observed in a subset of dopaminergic neurons in the Parkinson’s disease (PD)‐Lewy body dementia (LBD) model and a mouse model of aging.

**Conclusion:**

The consistent findings in multiple disease models validated the robust relationship between the cell cycle and senescence events in neurons. The multi‐model analysis conducted in multiple independent datasets demonstrated the applicability and effectiveness of our bioinformatics approach in a cross‐species setting.

